# Breast Cancer Survivors’ Exercise Preferences Change During an Exercise Intervention and are associated with Post-Intervention Physical Activity

**DOI:** 10.21203/rs.3.rs-2488848/v1

**Published:** 2023-01-20

**Authors:** Erica Schleicher, Edward McAuley, Kerry S Courneya, Phillip Anton, Diane K. Ehlers, Siobhan M. Phillips, Nashira I Brown, Robert A. Oster, Dorothy Pekmezi, Laura Q Rogers

**Affiliations:** University of Alabama at Birmingham; University of Illinois at Urbana-Champaign; University of Alberta; Southern Illinois University Carbondale; Mayo Clinic; Northwestern University Feinberg School of Medicine; University of Alabama at Birmingham; O'Neal Comprehensive Cancer Center at UAB; University of Alabama at Birmingham; O'Neal Comprehensive Cancer Center at UAB

**Keywords:** Breast Cancer Survivors, Exercise, Cancer, Survivorship, Oncology, Quality of Life, Preferences, Health Behavior

## Abstract

**Purpose:**

Exercise program preferences are important for designing physical activity (PA) interventions; yet may change following an intervention. Further, the relationship between preferences and PA behavior change is unclear. This study evaluated exercise program preferences among breast cancer survivors (BCS) before and after a behavioral intervention and associations between program preferences and PA change.

**Methods:**

BCS were randomized to the BEAT Cancer intervention (n = 110) or written materials (n = 112). Questionnaires assessed exercise program preferences. Minutes per week of moderate-to-vigorous PA (MVPA) were accelerometer-measured and self-reported at baseline (M0), post-intervention (M3), and 3-month follow-up (M6).

**Results:**

At M0, the majority of intervention group participants preferred exercising with others (62%) yet shifted to preferring exercising alone (59%) at M3 (*p* < 0.001). Furthermore, preferring exercising with others at M0 was associated with greater increases in self-reported MVPA between M0 and M6 (124.2 ± 152 vs. 53.1 ± 113.8, p = 0.014). BCS preferring facility-based exercise decreased after the BEAT Cancer intervention (14% vs. 7%, *p* = 0.039) and preferring exercising at home/had no preference at M0 had greater improvements in accelerometer-measured MVPA from M0 to M3 (74.3 ± 118.8 vs. −2.3 ± 78.4, p = 0.033) and M0 to M6 (44.9 ± 112.8 vs. 9.3 ± 30.4, p = 0.021). Exercise program preferences regarding mode of counseling, training supervision, and type of exercise changed from M0 to M3 but were not associated with changes in MVPA.

**Conclusion:**

Findings suggest BCS exercise program preferences may change after an intervention and be associated with changes in MVPA. Understanding the role of PA preferences will better inform the design and success of PA behavior change interventions.

## Background

As the number of women living beyond their breast cancer diagnosis continues to increase due to advancements in treatment [1], survivors often incur side effects and symptom burden from treatments, such as chemotherapy, that impact their quality of life [2]. Despite strong evidence of physical, psychological, and functional benefits from physical activity [3-8], breast cancer survivors are not participating in the recommended, 150 + minutes of moderate-to-vigorous physical activity per week (MVPA) [3, 8, 9]. In an effort to increase physical activity within the breast cancer survivor population, factors that may improve engagement in regular physical activity are being investigated, including programming preferences that could potentially be used to tailor behavioral change physical activity interventions.

Exercise programming preferences have been assessed within various cancer populations [10-12] and more specifically breast cancer survivors [13, 14]. However, the majority of these studies in cancer survivors have been cross-sectional, with very few longitudinal studies involving an exercise or physical activity intervention and none comparing preference changes in an intervention vs. control group [12]. This is a notable gap in the scientific literature because such preferences may change after engaging in a physical activity intervention [11,15], which could potentially impact longer term physical activity behavior maintenance.

The only two available reports of pre/post intervention change in exercise program preferences within the cancer population suggest that changes in preferences regarding location, social support, supervision, trainer education and counseling source may occur while other findings were inconsistent between the two studies [11, 15]. Head and neck cancer survivors recruited into a 12-week group resistance training intervention reported a change in preferred exercise counseling source, i.e., changed from preferring a personal trainer at baseline to preferring an instructor with experience working with cancer survivors following the intervention [11]. In contrast, women with metastatic breast cancer preferred their exercise counseling source to be a physical activity specialist before and after a 6 month home-based, unsupervised, aerobic training intervention [15]. However, both studies saw changes in preferred location and social support in which, after the interventions, participants switched to preferring exercising at a facility and with others who have a similar cancer diagnosis. It is important to note that these studies were not conducted within the non-metastatic breast cancer survivor population and implemented different types of physical activity behavior change interventions. Moreover, no prior study to our knowledge has compared preference changes in an intervention vs. control group, which is particularly important to account for natural evolution of preferences that may occur over time [16]. Therefore, our study purpose was to examine breast cancer survivors’ exercise program preferences before and after the 3-month BEAT Cancer physical activity behavior change intervention, which was comprised of 12 supervised exercise sessions tapered over six weeks to exclusively home-based sessions and six social cognitive theory-based exercise counseling sessions. By doing so, we can gain a better understanding of preference changes and how such changes relate to physical activity behavior post-intervention.

Based on the BEAT Cancer intervention design, we hypothesized breast cancer survivors would change their exercise program preferences following the BEAT Cancer intervention, and those who preferred programs that were home-based and unsupervised would have a greater increase in physical activity levels following the BEAT Cancer intervention.

## Methods

### Study Design

This is a secondary analysis of the multicenter two arm randomized controlled BEAT Cancer trial [7, 8]. Eligible participants were women, ages 18–70 years old with a history of ductal carcinoma in situ (DCIS) or stage I-IIIA breast cancer, and with a self-reported sedentary lifestyle (engaging in ≤ 30 minutes of vigorous or ≤ 60 minutes of moderate intensity physical activity per week on average over six months). Eligible women must have completed primary cancer treatment, been ≥ 8 weeks post-surgery, English speaking, and received medical clearance from their physician. Exclusion criteria included conditions that contraindicated physical activity or would interfere with assessments, and current participation in another exercise study. Additional details regarding inclusion and exclusion criteria and study design were reported by Rogers et al [8, 17]. Participants were recruited into the study between 2010 to 2013. Institutional Review Board (IRB) approval was obtained, and all participants provided written informed consent. Study personnel completing the assessments were blinded to the randomly assigned group allocation.

### Beat Cancer Intervention

This 3-month, social cognitive theory-based intervention consisted of 12 supervised exercise sessions tapered over six weeks, followed by three face-to-face exercise counseling sessions every two weeks with an exercise specialist. The exercise prescription aimed to gradually engage participants in 150 weekly minutes of MVPA using a progression previously reported [7]. Additionally, six group sessions led by trained facilitators provided behavioral counseling (time management, stress management, behavioral modification strategies etc.). An educational notebook with exercise-specific information related to intervention goals, exercise safety, nutrition information, exercise log sheets and personal heart rate monitor were provided to participants as part of the intervention. Further details about participant adherence and quality control for fidelity were previously published [7, 8, 17].

### Usual Care Intervention

The usual care (UC) group received written materials from the American Cancer Society describing physical activity recommendations for cancer survivors (see Rogers et al. [7] for additional details regarding the UC group intervention).

### Data Collection

Measurements were taken at baseline (M0), immediately post-intervention (M3) and 3 months after intervention completion (M6). Anthropometric measurements were obtained from a scale and stadiometer (Continental Health-O-Meter #400 DML medical scale [precision to nearest 8th of a pound] and Seca 763 Digital Column Scale [precision to nearest 0.1 lb]). Body mass index (BMI) was calculated from the height and weight [weight (kg)/height (m^2^)]. Demographics, medical history, and cancer history were self-reported. Exercise program preferences were collected via self-administered surveys based on previous work [13, 18, 19] (Table 1). Participants were asked: whom would you most like to receive exercise counseling? (cancer exercise specialist, personal trainer, health club trainer, cancer survivor, physician, nurse, no preference); how would you prefer to receive exercise counseling sessions? (one-on-one, group); and how would you most like to receive exercise counseling? (face-to-face, telephone, video tape, written material, internet, audiotape, interactive workbook, no preference). Additionally, participants were asked about their preferences regarding exercise training related social support (group, with others); trainer supervision (supervised, unsupervised, no preference); location (outdoors, at home, health club, cancer center, at work, no preference); program type (aerobic, resistance training, combination of both, no preference); exercise type (walking, resistance training, water activities, bicycling, yoga, pilates, jogging, other); and structure (scheduled, flexible, no preference).

For exercise programming, participants were asked: what is the most you would be willing to pay for an exercise program? ($0–10/month, $11–20/month, $21–30/month, $31–40+/month); what is the farthest you would be willing to travel to an exercise program? (0 miles, 1–15 miles, 16–30 miles, 31–45 + miles); and how far are you willing to travel to an exercise program if someone else paid for your gas costs? (0 miles, 1–15 miles, 16–30 miles, 31–45 + miles).

Physical activity was measured using ActiGraph accelerometers (models GT1M and GT3X; protocol previously published) [17]. Additionally, self-reported leisure time physical activity was assessed using the Godin Leisure-Time Exercise Questionnaire [20, 21]. The frequency and average duration of exercise bouts per week over the previous month at light, moderate, and vigorous intensity were collected, and minutes of moderate intensity physical activity were calculated. Both accelerometer and self-reported minutes of vigorous intensity activity were multiplied by two and added to moderate minutes.

### Statistical Analysis

Self-reported exercise program preferences were described by frequency and percentages at baseline and 3-months. To investigate the differences in program preferences before and immediately after the 3-month intervention period, preferences at M0 and M3 were dichotomized for both groups, and the McNemar chi-square test was used to examine changes between the two time periods for paired data. In addition, the Pearson chi-square test, or Fisher’s exact test (if the assumptions for the Pearson chi-square were not satisfied), was used for group comparisons (BEAT cancer group, UC group) of categorical variables. Associations between program preferences and change in MVPA were examined using the two-group *t* test. Statistical tests were two-sided. Statistical significance was set at *p* < 0.05. Analyses were performed using SPSS software, version 28 (IBM Corp., Chicago, IL).

## Results

### Participant Characteristics

A total of 222 breast cancer survivors were randomized to either the BEAT Cancer (n = 110) or UC (n =112) groups. The mean age of participants was 54 ± 9 years old, with the majority being White (84%). Participants had an average of 16 ± 3 years of education. Cancer stages varied from, DCIS (11%), stage I (42%), stage II (35%), and stage III (12%). The average time since breast cancer diagnosis was 54 ± 55 months, with 58% and 68% reporting a history of chemotherapy and radiation therapy, respectively. Approximately half (49%) of all participants reported current hormone therapy [7]. Detailed descriptions participant demographics have been published elsewhere [7, 8].

### Program Preferences At Baseline

Participant preferences for exercise counseling, training, and programming are provided in Table 1 (*see below*). At M0 for all participants combined, the most preferred exercise counseling source was a cancer exercise specialist (41%), with one-on-one sessions (67%) and face-to-face delivery (77%). Other preference options included exercising with others (57%), supervised (69%), and outdoors (28%). Although most breast cancer survivors preferred a combination of both aerobic and resistance training (67%), the most popular specific type of exercise was walking (54%). With regard to program structure, participants were most interested in a flexible rather than scheduled exercise program (42%). When asked to indicate the most they were willing to pay per month for an exercise program, 31% responded ≤$10, with 26% indicating $11-$20, 25% indicating $21-$30, and 18% indicating ≥$31. Traveling up to 15 miles was the furthest most participants were willing to travel for an exercise program (74%), even if gas were paid for by another entity (62%).

### Program Preference Changes Within Each Study Group

Within the BEAT Cancer group, five preferences had significant changes in the proportion of participants endorsing them from M0 to M3 (Table 2). A majority of BEAT Cancer participants preferred face-to-face delivery to other options (video tape, written materials, internet, or no preference) but this proportion decreased from M0 to M3 (77% vs. 65%, *p* = 0.019). At M0, the majority of participants preferred exercising with others (i.e., 62%); however, this decreased to 39% at M3 such that the majority (59%) preferred exercising alone at M3 (*p* < 0.001). The number of BEAT Cancer participants preferring supervised exercise training decreased from M0 to M3 (67% vs. 41%, *p* = 0.015). Furthermore, the number of participants who preferred to exercise at a facility decreased after the intervention (14% vs. 7%, *p* = 0.039). Although a combination of aerobic and resistance training was most preferred at M0 and M3 (83% vs. 69%, *p* = 0.011), aerobic and resistance training as separate preferences gained interest after the intervention. No other within group differences in program preferences were noted for the BEAT Cancer intervention group.

Within the UC group, the proportion of participants preferring a program characteristic significantly changed for only two preferences from M0 to M3 (Table 2). The number of participants who preferred their counseling from a source other than a cancer exercise specialist increased (62% vs. 72%, *p* =0.05). Similar to the BEAT Cancer group, the number of UC participants preferring supervised exercise training decreased from M0 to M3 (71% vs. 55%, *p* = 0.004). No other within group differences in program preferences were noted for the UC group.

#### Proportion of Participants Who Changed Their Preference from M0 to M3 for BEAT Cancer vs. Usual Care

When comparing the proportion of participants who changed their preference from M0 to M3 for BEAT Cancer vs. UC groups, a greater proportion of BEAT Cancer participants changed their preference for supervised or unsupervised exercise training compared to UC (54% vs. 46%, *p* = 0.02). The proportion of participants changing their preferences for other program characteristics were not statistically significantly different for the BEAT Cancer vs. UC between M0 and M3 (data not shown).

### Associations With Change In Physical Activity Within Beat Cancer Intervention

Baseline (M0) preferences related to training social support and location were found to be associated with changes in MVPA levels (Table 3). Within the BEAT Cancer group, greater increases in self-reported MVPA between M0 and M6 were observed in those who preferred training in a group setting at M0 (124.2 ± 152 vs. 53.1 ± 113.8, *p* = 0.014). Those who preferred exercising at home or had no preference at M0 had a greater improvement in accelerometer-measured MVPA from M0 to M3 (74.3 ± 118.8 vs. −2.3 ± 78.4, *p* = 0.033) and M0 to M6 (44.9 ± 112.8 vs. 9.3 ± 30.4, *p* = 0.021) than those who preferred exercising at a facility. In contrast, preferring to exercise at home or no preference rather than a facility after the BEAT Cancer intervention (i.e., at M3) was associated with a decline in self-reported MVPA from M3 to M6 (−34.3 ± 143.2, *p* = 0.044). No other M0 preferences were significantly associated with MVPA change from M0 to M3 or M6 nor were there any other M3 preferences associated with a change from M3 to M6 among the preferences with significant preference changes.

## Discussion

Breast cancer survivors’ exercise program preferences can change during an exercise intervention and may be associated with exercise behavior change from pre- to post-intervention. This is the first randomized intervention study to our knowledge to elucidate program preference changes for the intervention vs. control condition coupled with exploring the associations between these preferences and post-intervention MVPA. Statistically significant changes over the 3-month intervention period were noted among the BEAT Cancer intervention recipients for five preferences (i.e., delivery, group/alone, supervision, location, type), while only two of the thirteen program preferences (i.e., counseling source and supervision) demonstrated a statistically significant change among those in the UC group. Two baseline preferences (i.e., group/alone and location) were associated with the BEAT Cancer intervention success from M0 to M3 and from M0 to M6 in the BEAT Cancer intervention group. Yet, location was the only preference associated with change in physical activity during the 3-month post-intervention period (M3-M6).

After receiving the BEAT intervention, the proportion of participants interested in face-to-face exercise counseling declined; however, it was still the most preferred mode of delivery. Similarly, the proportion preferring supervised exercise training decreased when assessed immediately post-intervention. Although the same change was seen within the UC group, analyses indicated a greater proportion of BEAT Cancer participants changed their preference from supervised to unsupervised training when compared with UC. These results indicate survivors had less of a desire for professional exercise support following the BEAT Cancer intervention. This could be a result of the intervention design tapering the exercise sessions from supervised in a facility to unsupervised home-based sessions over the 3-month period with the goal of helping participants become more independent with their physical activity behaviors. It seems possible that this design gave the participants the education needed to feel more capable of exercising on their own without needing more professional support.

Also, fewer survivors were interested in exercising at a facility after participating in the intervention, which was expected as the intervention transitioned from facility to home-based exercise sessions over the intervention period. While home-based exercise gained interest, having this preference at M3 was associated with a decrease in self-reported MVPA following the intervention from M3 to M6. Yet, free-living accelerometer-measured MVPA levels did not decrease among those who preferred to exercise at home. A possible explanation for these results may be that without traveling to a designated exercise location, the participants may feel like they are not being as active, thus self-reporting lower activity levels. Further research is needed to better understand if behavior changes preferences or vice versa and how these relationships may differ for free-living vs. volitional leisure-time physical activity behavior. Survivors who preferred exercise training in a group setting at M0 exhibited greater increase in self-reported MVPA between M0 and M6; however, at M3 the majority changed to preferring exercise training alone. As seen in this study and previous studies, having social support by training with a partner or in a group setting can provide accountability and increase activity levels [22-24]; yet, coordinating exercise in a group setting may be more difficult and require more effort than exercising alone, which could have contributed to this preference change. Since the intervention tapered to individual exercises sessions at home the participants may have gained confidence in exercising on their own. The majority of participants preferred a combination of both aerobic and resistance exercise training before and after the BEAT cancer intervention, yet after the intervention a portion of participants changed to no longer preferring the combination of both, enough to be statistically significant. Furthermore, when asked about specific types of exercise, resistance exercise training was least preferred by the majority of participants who reported to prefer another form of exercise (i.e., walking, water activities, cycling, yoga, etc.) over resistance training. Since the BEAT Cancer intervention focused on aerobic exercise training, specifically walking, participants may have been more inclined to focus strictly on aerobic rather than resistance exercise.

These results are consistent with those reported by Owens et al. [25] examining exercise program preferences in adults with metabolic syndrome. Participants’ preferences changed significantly from preferring supervised exercise training in a group setting to favoring independent (i.e., alone), unsupervised training following a 16-week, community-based lifestyle intervention. Our study and Owens et al. demonstrated a shift in program preferences from having a desire for professional supervised training and social support to wanting unsupervised, independent training sessions. Intervention fatigue was suggested as an explanation for the decreased interest in support, which could be a factor within any population, including breast cancer survivors. Alternatively, it was previously reported by our group that the BEAT Cancer intervention significantly improved self-efficacy within this sample [26], thus participants may have gained more confidence in exercising after the intervention and required less support.

Contrasting results were found in women with metastatic breast cancer following a 6-month unsupervised home-based intervention. Most participants favored training at home but following the unsupervised home-based intervention, the majority changed to preferring training at a community-based facility. Further, patients with head and neck cancer had a significant increase in preference towards training at a cancer center following a 12-week, group resistance exercise training intervention. While the findings are not consistent with our results, this preference change could support intervention fatigue, as the observed preference changes were a shift away from the intervention design. It seems possible that these contrasting results could be due to people preferring variety and influence the desire for the opposite of the intervention.

No statistically significant differences were found for counseling source preference in this study; however, previous studies in the cancer patient populations found a statistically significant change [11, 15]. The majority of head and neck patients changed from preferring a personal trainer to a professional with a kinesiology degree [11], while the majority of metastatic breast cancer patients preferred an exercise specialist with an increase in this preference following their intervention [15]. While both studies can offer potential explanations for the observed results, it is important to note that metastatic breast and head and neck cancer patients have greater risks for side effects or adverse events and cancer/treatment-related sequelae, which may influence the differences in results.

The strengths of this study include the randomized controlled trial design, large sample size (relative to the current literature), high participant retention rate, and assessment of both objective and self-report physical activity within the context of statistically significant intervention efficacy at M3 and M6 [7, 11, 15, 25]. This study adds value to the current limited literature reporting pre/post changes in program preferences during an exercise intervention in cancer survivors. Further, no prior study to our knowledge within the cancer population has compared program preference changes in an intervention vs. control group, in an effort to account for natural evolution of preferences that may occur over time in the absence of an intervention [25].

### Study Limitations

We acknowledge this study is limited in variability among race and rurality of the sample. Participants were primarily White women residing in metro or small urban areas and may have better access to exercise resources than non-White breast cancer survivors in more rural or inner-city urban areas. Additionally, these findings may not be generalizable to other cancer types beyond breast or to resistance training interventions. Further, our findings should not be used to conclude the prevalence of exercise preferences because it was not a population-based survey. Nevertheless, comparison of our study with a similar survey conducted in a large population-based sample of breast cancer survivors [14], demonstrates similar findings in that both report the majority of breast cancer survivors preferring face-to-face exercise counseling, flexible exercise scheduling, walking as the exercise type, and exercising alone, unsupervised, and at home/no preference [14].

### Clinical Implications

Our study suggests that tailoring to social support (i.e., group vs. individual) and location (i.e., home-based vs. facility) preferences may be the key for increasing perceived acceptability of and engagement with an intervention. Further, preference may change over time suggesting periodic reassessment for additional tailoring may be indicated. Future study is warranted to determine why preferences change and whether tailoring an intervention towards preferences is feasible and optimizes exercise behavior change short and long term. Current approaches in the exercise oncology field acknowledge that a generic exercise program does not work for the general population and interventions are commonly tailored using the Frequency, Intensity, Time, Type (FITT) principles and no other intervention characteristics. Previous research has highlighted that tailoring interventions solely using the FITT principles may not be enough to optimize adherence to a physical activity intervention within the breast cancer population [27]. It is important to note the results in the present study demonstrate that breast cancer survivors have specific exercise program preferences and could benefit from interventions that are tailored using preferences along with the FITT principles.

## Conclusion

The main goal of the current study was to gain a better understanding of program preference changes during an exercise behavior change intervention vs. UC and the associations between preferences and breast cancer survivors’ physical activity levels post-intervention. In summary, several preferences changed during the exercise intervention primarily mirroring (or better aligning with) the intervention design with a few, albeit limited, number of preferences being associated with physical activity outcomes.

## Figures and Tables

**Table 1:** Program preferences before and after the BEAT Cancer intervention overall and by study group.

Variable	Overall (*n* = 218) n (%)		BEAT Cancer (*n* = 106) n (%)		Usual Care (*n* = 112) n (%)	
	M0	M3	M0	M3	M0	M3
If you were interested in exercising on a regular basis this week, from whom would you most like to receive exercise counseling?						
Cancer exercise specialist	90 (41)	82 (38)	47 (44)	51 (48)	43 (38)	31 (28)
Personal trainer	51 (23)	45 (21)	27 (26)	19 (18)	24 (21)	26 (23)
Health club trainer	23 (11)	25 (12)	8 (8)	7 (7)	15 (13)	18 (16)
Cancer survivor	1 (1)	4 (2)	0 (0)	2 (2)	1 (1)	2 (2)
Physician	0 (0)	3 (1)	0 (0)	0 (0)	0 (0)	3 (3)
Nurse	0 (0)	1 (1)	0 (0)	1 (1)	0 (0)	0 (0)
No preference	53 (24)	58 (27)	24 (23)	26 (25)	29 (26)	32 (29)
How would you prefer to receive exercise counseling sessions?						
One-on-one	145 (67)	145 (67)	64 (60)	69 (65)	81 (74)	76 (69)
Group	71 (33)	71 (33)	42 (40)	37 (35)	29 (26)	34 (31)
If you were interested in exercising on a regular basis this week, how would you most like to receive exercise counseling?						
Face-to-face	167 (77)	146 (67)	82 (77)	69 (65)	85 (76)	77 (69)
Telephone	2 (1)	2 (1)	2 (2)	2 (2)	0 (0)	0 (0)
Video tape	8 (4)	15 (7)	2 (2)	6 (6)	6 (5)	9 (8)
Written material	4 (2)	7 (3)	1 (1)	4 (4)	3 (3)	3 (3)
Internet	5 (2)	6 (3)	2 (2)	3 (3)	3 (3)	3 (3)
Audiotape	0 (0)	1 (1)	0 (0)	0 (0)	0 (0)	1 (1)
Interactive workbook	1 (1)	0 (0)	1 (1)	0 (0)	0 (0)	0 (0)
No preference	31 (14)	41 (19)	16 (15)	22 (21)	15 (13)	19 (17)
How would you prefer to exercise?						
Alone	93 (43)	120 (56)	40 (39)	61 (59)	53 (48)	59 (53)
With others	122 (57)	95 (44)	64 (62)	43 (41)	58 (52)	52 (47)
How would you prefer to perform exercise this week?						
Supervised	151 (69)	105 (48)	71 (67)	43 (41)	80 (71)	62 (55)
Unsupervised	13 (6)	53 (24)	7 (7)	38 (36)	6 (5)	15 (13)
No preference	54 (25)	60 (28)	28 (26)	25 (24)	26 (23)	35 (31)
If you were going to do some kind of regular exercise or are now exercising, where would you most like to do that exercise on a regular basis this week?						
Outdoors	60 (28)	71 (33)	28 (26)	38 (36)	32 (29)	33 (30)
At home	42 (19)	61 (28)	20 (19)	36 (34)	22 (20)	25 (22)
In a health club	42 (19)	46 (21)	15 (14)	15 (14)	27 (24)	31 (28)
At a cancer center	28 (13)	17 (8)	15 (14)	7 (7)	13 (12)	10 (9)
At work	2 (1)	3 (1)	0 (0)	1 (1)	2 (2)	2 (2)
Other	11 (5)	12 (6)	7 (7)	7 (7)	4 (4)	5 (5)
No preference	33 (15)	8 (4)	21 (20)	2 (2)	12 (11)	6 (5)
If you were to participate in an exercise program this week, which would you prefer?						
Aerobic exercise	22 (10)	40 (18)	12 (11)	25 (24)	10 (9)	15 (13)
Resistance training	14 (6)	22 (10)	6 (6)	8 (8)	8 (7)	14 (13)
Aerobic & resistance training	145 (67)	133 (61)	69 (65)	65 (61)	76 (68)	68 (61)
No preference	37 (17)	23 (11)	19 (18)	8 (8)	18 (16)	15 (13)
What type of exercise would you most prefer to do this week?						
Walking	117 (54)	121 (56)	53 (50)	69 (65)	64 (57)	52 (46)
Resistance training	33 (15)	26 (12)	17 (16)	8 (8)	16 (14)	18 (16)
Water activities	24 (11)	25 (12)	12 (11)	12 (11)	12 (11)	13 (12)
Bicycling	12 (6)	12 (6)	6 (6)	4 (4)	6 (5)	8 (7)
Yoga	11 (5)	14 (6)	8 (8)	7 (7)	3 (3)	7 (6)
Pilates	7 (3)	4 (2)	4 (4)	1 (1)	3 (3)	3 (3)
Jogging	4 (2)	5 (2)	1 (1)	2 (2)	3 (3)	3 (3)
Other	10 (5)	11 (5)	5 (5)	3 (3)	5 (5)	8 (7)
What would you prefer the structure of your exercise to be?						
Flexible	90 (41)	115 (53)	47 (44)	59 (56)	43 (38)	56 (50)
Scheduled	92 (42)	79 (36)	44 (42)	38 (36)	48 (43)	41 (37)
No preference	36 (17)	24 (11)	15 (14)	9 (9)	21 (19)	15 (13)
What is the most you are willing to pay for an exercise program?						
$0 per month	26 (12)	33 (15)	14 (13)	15 (14)	12 (11)	18 (16)
$1-$10 per month	42 (19)	28 (13)	21 (20)	14 (13)	21 (19)	14 (13)
$11-$20 per month	57 (26)	62 (28)	23 (22)	30 (28)	34 (30)	32 (29)
$21-$30 per month	54 (25)	41 (19)	25 (24)	21 (20)	29 (26)	20 (18)
$31-$40 per month	20 (9)	33 (15)	11 (10)	15 (14)	9 (8)	18 (16)
$40 ≥ per month	19 (9)	21 (10)	12 (11)	11 (10)	7 (6)	10 (9)
What is the farthest you are willing to travel to an exercise program?						
0 Miles	8 (4)	12 (6)	3 (3)	6 (6)	5 (5)	6 (5)
1–15 Miles	151 (70)	126 (75)	75 (71)	74 (71)	76 (68)	88 (79)
16–30 Miles	45 (21)	34 (16)	23 (22)	20 (19)	22 (20)	14 (13)
31–45 Miles	12 (6)	8 (4)	4 (4)	5 (5)	8 (7)	3 (3)
46–60 Miles	1 (1)	1 (1)	0 (0)	0 (0)	1 (1)	1 (1)
60 > Miles	0 (0)	0 (0)	0 (0)	0 (0)	0 (0)	0 (0)
How far are you willing to travel to an exercise program if someone else paid for your gas costs?						
0 Miles	25 (12)	10 (5)	11 (11)	5 (5)	14 (13)	5 (5)
1–15 Miles	109 (50)	129 (60)	53 (51)	59 (56)	56 (50)	70 (63)
16–30 Miles	55 (25)	54 (25)	30 (29)	29 (28)	25 (22)	25 (22)
31–45 Miles	18 (8)	17 (8)	5 (5)	8 (8)	13 (12)	9 (8)
46–60 Miles	4 (2)	4 (2)	1 (1)	2 (2)	3 (3)	2 (2)
60 ≥ Miles	6 (3)	3 (1)	5 (5)	2 (2)	1 (1)	1 (1)

M0- Month 0, Baseline

M3- Month 3, Immediately after intervention

M6- Month 6, follow-up after intervention completion

**Table 2:** Dichotomized program preferences pre-and post-BEAT Cancer intervention by group (binomial distribution was used) (n (%)).

Variable	BEAT Cancer (*n* = 106)		Usual Care (*n* = 112)	
	M0	M3	M0	M3
If you were interested in exercising on a regular basis this week, from whom would you most like to receive exercise counseling?				
Cancer exercise specialist	47 (44)	51 (48)	43 (38)	31 (28)
Other	59 (56)	55 (52)	69 (62)	81 (72)*
How would you prefer to receive exercise counseling sessions?				
One-on-One	64 (60)	69 (65)	81 (74)	76 (69)
Group	42 (40)	37 (35)	29 (26)	34 (31)
If you were interested in exercising on a regular basis this week, how would you most like to receive exercise counseling?				
Face-to-face	82 (77)	69 (65)*	85 (76)	77 (69)
Other	24 (23)	37 (35)	27 (24)	35 (31)
How would you prefer to exercise?				
Alone	40 (39)	61 (59)*	53 (48)	59 (53)
With Others	64 (62)	43 (41)	58 (52)	52 (47)
How would you prefer to perform exercise this week?				
Supervised	71 (67)	43 (41)	80 (71)	62 (55)*
Unsupervised	35 (33)	63 (59)*	32 (29)	50 (45)
If you were going to do some kind of regular exercise or are now exercising, where would you most like to do that exercise on a regular basis this week?				
At Home/ No preference	91 (86)	99 (93)*	99 (88)	102 (91)
Facility	15 (14)	7 (7)	13 (12)	10 (9)
If you were to participate in an exercise program this week, which would you prefer?				
Aerobic or strength training	18 (17)	33 (31)	18 (16)	29 (26)
Both/ No preference	88 (83)	73 (69)*	94 (84)	83 (74)
What type of exercise would you most prefer to do this week?				
Walking	65 (60)	62 (57)	53 (47)	61 (55)
Other	44 (40)	47 (43)	59 (53)	51 (46)
What type of exercise would you most prefer to do this week?				
Strength training	22 (20)	19 (17)	30 (27)	25 (22)
Other	87 (80)	90 (83)	82 (73)	87 (78)
What would you prefer the structure of your exercise to be?				
Flexible	62 (59)	68 (64)	64 (57)	71 (63)
Scheduled	44 (42)	38 (36)	48 (43)	41 (37)
What is the most you are willing to pay for an exercise program?				
$0-$20	58 (55)	59 (56)	67 (60)	64 (57)
$21-$40+	48 (45)	47 (44)	45 (40)	48 (43)
What is the farthest you are willing to travel to an exercise program?				
0–15 Miles	78 (74)	80 (76)	81 (72)	94 (84)
16–45 +Miles	27 (26)	25 (24)	31 (28)	18 (16)
How far are you willing to travel to an exercise program if someone else paid for your gas costs?				
0–15 Miles	64 (61)	64 (61)	70 (63)	75 (67)
16–45 +Miles	41 (39)	41 (39)	42 (38)	37 (33)

* indicate p-value < 0.05

M0- Month 0, Baseline

M3- Month 3, Immediately after intervention

M6- Month 6, follow-up after intervention completion

**Table 3:** Statistically significant associations between program preferences and physical activity levels within the BEAT Cancer group (n = 102)

Baseline Exercise Program Preferences	Mean difference in MVPA levels between timepoints Mean (SD)	
*Accelerometer-measured MVPA*	*M0-M3*	*p value*
Exercise Training Location		
At Home/ No preference	74.31 (118.75)	0.033
Facility	−2.25 (78.36)	
*Accelerometer-measured MVPA*	*M0-M6*	*p value*
Exercise Training Location		
At Home/ No preference	44.85 (112.81)	0.021
Facility	9.25 (30.37)	
*Self-Reported MVPA*	*M0-M6*	*p value*
Exercise Training Social Support		
Alone	53.08 (113.76)	0.014
With Others	124.23 (152.04)	
M3 Exercise Program Preferences	Mean difference in MVPA levels between timepoints Mean (SD)	
*Self-Reported MVPA*	*M3-M6*	*p value*
Exercise Training Location		
At Home/ No preference	−34.34 (143.15)	0.044
Facility	79 (125.81)	

MVPA- Moderate-to-vigorous physical activity

SD- Standard deviation

M0- Month 0, Baseline

M3- Month 3, Immediately after intervention

M6- Month 6, follow-up after intervention completion

## Abbreviations

BCS: Breast Cancer Survivor
BEAT: Cancer Better Exercise Adherence after Treatment for Cancer
BMI: Body Mass Index
DCIS: Ductal Carcinoma in Situ
FITT: Frequency, Intensity, Time, Type
IRB: Institutional Review Board
MVPA: Moderate-to-vigorous Physical Activity
PA: Physical Activity
UC: Usual Care

## References

[1] Siegel RL, Miller KD, Fuchs HE, Jemal A: Cancer Statistics, 2021. CA Cancer J Clin 2021, 71(1):7–33. 33433946 10.3322/caac.21654

[2] Wu HS, Harden JK: Symptom burden and quality of life in survivorship: a review of the literature. Cancer Nurs 2015, 38(1):E29–54. 24831042 10.1097/NCC.0000000000000135

[3] Campbell KL, Winters-Stone KM, Wiskemann J, May AM, Schwartz AL, Courneya KS, Zucker DS, Matthews CE, Ligibel JA, Gerber LH Exercise Guidelines for Cancer Survivors: Consensus Statement from International Multidisciplinary Roundtable. Medicine and science in sports and exercise 2019, 51(11):2375–2390. 31626055 10.1249/MSS.0000000000002116 PMC8576825

[4] Cormie P, Zopf EM, Zhang X, Schmitz KH: The Impact of Exercise on Cancer Mortality, Recurrence, and Treatment-Related Adverse Effects. Epidemiol Rev 2017, 39(1):71–92. 28453622 10.1093/epirev/mxx007

[5] Hong F, Ye W, Kuo CH, Zhang Y, Qian Y, Korivi M: Exercise Intervention Improves Clinical Outcomes, but the "Time of Session" is Crucial for Better Quality of Life in Breast Cancer Survivors: A Systematic Review and Meta-Analysis. Cancers (Basel) 2019, 11(5). 10.3390/cancers11050706 PMC6562879 31121916

[6] Mishra SI, Scherer RW, Geigle PM, Berlanstein DR, Topaloglu O, Gotay CC, Snyder C: Exercise interventions on health-related quality of life for cancer survivors. Cochrane Database Syst Rev 2012, 2012(8):Cd007566. 22895961 10.1002/14651858.CD007566.pub2 PMC7387117

[7] Rogers LQ, Courneya KS, Anton PM, Hopkins-Price P, Verhulst S, Vicari SK, Robbs RS, Mocharnuk R, McAuley E: Effects of the BEAT Cancer physical activity behavior change intervention on physical activity, aerobic fitness, and quality of life in breast cancer survivors: a multicenter randomized controlled trial. Breast cancer research and treatment 2015, 149(1):109–119. 25417174 10.1007/s10549-014-3216-z PMC4435784

[8] Rogers LQ, Courneya KS, Carter SJ, Anton PM, Verhulst S, Vicari SK, Robbs RS, McAuley E: Effects of a multicomponent physical activity behavior change intervention on breast cancer survivor health status outcomes in a randomized controlled trial. Breast cancer research and treatment 2016, 159(2):283–291. 27539586 10.1007/s10549-016-3945-2 PMC5014640

[9] Gavin KL, Welch WA, Conroy DE, Kozey-Keadle S, Pellegrini C, Cottrell A, Nielsen A, Solk P, Siddique J, Phillips SM: Sedentary behavior after breast cancer motivational, demographic, disease, and health status correlates of sitting time in breast cancer survivors. Cancer Causes Control 2019, 30(6):569–580. 30919252 10.1007/s10552-019-01153-7 PMC6538257

[10] Belanger LJ, Plotnikoff RC, Clark A, Courneya KS: A survey of physical activity programming and counseling preferences in young-adult cancer survivors. Cancer Nurs 2012, 35(1):48–54. 21558852 10.1097/NCC.0b013e318210220a

[11] Jackson C, Dowd AJ, Capozzi LC, Bridel W, Lau HY, Culos-Reed SN: A turning point: Head and neck cancer patients' exercise preferences and barriers before and after participation in an exercise intervention. Eur J Cancer Care (Engl) 2018, 27(2):e12826. 29377317 10.1111/ecc.12826

[12] Wong JN, McAuley E, Trinh L: Physical activity programming and counseling preferences among cancer survivors: a systematic review. Int J Behav Nutr Phys Act 2018, 15(1):48. 29879993 10.1186/s12966-018-0680-6 PMC5992647

[13] Rogers LQ, Courneya KS, Verhulst S, Markwell SJ, McAuley E: Factors associated with exercise counseling and program preferences among breast cancer survivors. J Phys Act Health 2008, 5(5):688–705. 18820344 10.1123/jpah.5.5.688

[14] Rogers LQ, Markwell SJ, Verhulst S, McAuley E, Courneya KS: Rural breast cancer survivors: exercise preferences and their determinants. Psycho-Oncology 2009, 18(4):412–421. 19241491 10.1002/pon.1497

[15] Delrieu L, Vallance JK, Morelle M, Fervers B, Pialoux V, Friedenreich C, Dufresne A, Bachelot T, Heudel P-E, Trédan O : Physical activity preferences before and after participation in a 6-month physical activity intervention among women with metastatic breast cancer. European Journal of Cancer Care 2020, 29(1):e13169. 31571315 10.1111/ecc.13169

[16] Job JR, Eakin EG, Reeves MM, Fjeldsoe BS: Evaluation of the Healthy Living after Cancer text message-delivered, extended contact intervention using the RE-AIM framework. BMC Cancer 2021, 21 (1):1081. 34620115 10.1186/s12885-021-08806-4 PMC8496009

[17] Rogers LQ, McAuley E, Anton PM, Courneya KS, Vicari S, Hopkins-Price R Verhulst S, Mocharnuk R, Hoelzer K: Better exercise adherence after treatment for cancer (BEAT Cancer) study: rationale, design, and methods. Contemporary clinical trials 2012, 33(1):124–137. 21983625 10.1016/j.cct.2011.09.004 PMC3253876

[18] Rogers LQ, Courneya KS, Shah P, Dunnington G, Hopkins-Price P: Exercise stage of change, barriers, expectations, values and preferences among breast cancer patients during treatment: a pilot study. Eur J Cancer Care (Engl) 2007, 16(1):55–66. 17227354 10.1111/j.1365-2354.2006.00705.x

[19] Jones LW, Courneya KS: Exercise counseling and programming preferences of cancer survivors. Cancer Pract 2002, 10(4):208–215. 12100105 10.1046/j.1523-5394.2002.104003.x

[20] Godin G, Jobin J, Bouillon J: Assessment of leisure time exercise behavior by self-report: a concurrent validity study. Can J Public Health 1986, 77(5):359–362. 3791117

[21] Godin G, Shephard RJ: A simple method to assess exercise behavior in the community. Canadian journal of applied sport sciences Journal canadien des sciences appliquees au sport 1985, 10(3):141–146. 4053261

[22] Leach HJ, Covington KR, Voss C, LeBreton KA, Harden SM, Schuster SR: Effect of Group Dynamics-Based Exercise Versus Personal Training in Breast Cancer Survivors. Oncol Nurs Forum 2019, 46(2):185–197. 30767964 10.1188/19.ONF.185-197

[23] Rogers LQ, Markwell SJ, Verhulst S, McAuley E, Courneya KS: Rural breast cancer survivors: exercise preferences and their determinants. Psychooncology 2009, 18(4:412–421. 19241491 10.1002/pon.1497

[24] Winters-Stone KM, Lyons KS, Dobek J, Dieckmann NF, Bennett JA, Nail L, Beer TM: Benefits of partnered strength training for prostate cancer survivors and spouses: results from a randomized controlled trial of the Exercising Together project. J Cancer Surviv 2016, 10(4):633–644. 26715587 10.1007/s11764-015-0509-0

[25] Owen K, Pettman T, Haas M, Viney R, Misan G: Individual preferences for diet and exercise programmes: changes over a lifestyle intervention and their link with outcomes. Public Health Nutr 2010, 13(2):245–252. 19656436 10.1017/S1368980009990784

[26] Rogers LQ, Courneya KS, Anton PM, Hopkins-Price R Verhulst S, Robbs RS, Vicari SK, McAuley E: Social Cognitive Constructs Did Not Mediate the BEAT Cancer Intervention Effects on Objective Physical Activity Behavior Based on Multivariable Path Analysis. Ann Behav Med 2017, 51 (2):321–326. 27752993 10.1007/s12160-016-9840-6 PMC5373935

[27] Kirkham AA, Bonsignore A, Bland KA, McKenzie DC, Gelmon KA, CL VANP, Campbell KL: Exercise Prescription and Adherence for Breast Cancer One Size Does Not FITT All. Medicine and science in sports and exercise 2018, 50(2):177–186. 28991038 10.1249/MSS.0000000000001446

